# Effects of chondroitin sulfate proteoglycan 4 (NG2/CSPG4) on soft-tissue sarcoma growth depend on tumor developmental stage

**DOI:** 10.1074/jbc.M117.805051

**Published:** 2017-12-01

**Authors:** Shu-Hsuan Claire Hsu, Puviindran Nadesan, Vijitha Puviindran, William B. Stallcup, David G. Kirsch, Benjamin A. Alman

**Affiliations:** From the ‡ Department of Orthopaedic Surgery and RegenerationNext Initiative and; the ¶ Department of Radiation Oncology, Duke University, Durham, North Carolina 27710 and; the § Tumor Microenvironment and Cancer Immunology Program, Cancer Center, Sanford Burnham Prebys Medical Discovery Institute, La Jolla, California 92037

**Keywords:** cell proliferation, chondroitin sulfate, gene expression, insulin-like growth factor (IGF), osteosarcoma, transgenic mice

## Abstract

Sarcomas, and the mesenchymal precursor cells from which they arise, express chondroitin sulfate proteoglycan 4 (NG2/CSPG4). However, NG2/CSPG4's function and its capacity to serve as a therapeutic target in this tumor type are unknown. Here, we used cells from human tumors and a genetically engineered autochthonous mouse model of soft-tissue sarcomas (STSs) to determine NG2/CSPG4's role in STS initiation and growth. Inhibiting NG2/CSPG4 expression in established murine and human STSs decreased tumor volume by almost two-thirds and cell proliferation rate by 50%. NG2/CSPG4 antibody immunotherapy in human sarcomas established as xenografts in mice similarly decreased tumor volume, and expression of a lentivirus blocking *NG2/CSPG4* expression inhibited tumor cell proliferation and increased the latency of engraftment. Gene profiling showed that *Ng2/Cspg4* deletion altered the expression of genes regulating cell proliferation and apoptosis. Surprisingly, *Ng2/Cspg4* deletion at the time of tumor initiation resulted in larger tumors. Gene expression profiling indicated substantial down-regulation of insulin-like growth factor binding protein (*Igfbp*) genes when *Ng2/Cspg4* is depleted at tumor initiation, but not when *Ng2/Cspg4* is depleted after tumor initiation. Such differences may have clinical significance, as therapeutic targeting of a signaling pathway such as NG2/CSPG4 may have different effects on cell behavior with tumor progression. NG2/CSPG4 depletion has divergent effects, depending on the developmental stage of sarcoma. In established tumors, IGF signaling is active, and NG2 inhibition targets cell proliferation and apoptosis.

## Introduction

Soft-tissue sarcomas are malignant tumors arising in mesenchymal derived tissues that are classified into subtypes primarily based on histology (1). Undifferentiated pleomorphic sarcoma is the most common and aggressive subtype in adults, with a roughly 50% 5-year survival rate (1–4). This sarcoma type displays a highly heterogeneous and undifferentiated histological phenotype, which suggests that it may represent an end point for other sarcomas that have undergone dedifferentiation from a cell initially of mesenchymal origin (5, 6).

Chondroitin sulfate proteoglycan (CSPG4), also called neural-glia 2 (NG2) is a membrane protein expressed by progenitor mesenchymal cells (*e.g.* pericytes), immature keratinocytes, melanocytes, and cells in several tumor types (7). As a gene expressed by mesenchymal progenitors, its expression could play a role in sarcoma initiation. It is a transmembrane protein that can potentiate the activities of other signaling-transducing systems, such as integrin and MAPK signaling pathways (8–10). NG2/CSPG4 can bind to and present growth factors (*e.g.* basic fibroblast growth factor and platelet-derived growth factor) to their cognate receptor tyrosine kinase receptors (11, 12). In human glioblastoma cells, NG2/CSPG4-mediated activation of integrin signaling promotes cell survival through sustained activation of Akt (protein kinase B) (13, 14) and chemoresistance through integrin-dependent PI3K/Akt signaling (8). In human melanomas, NG2/CSPG4 functions to activate the MEK/ERK1/2 pathway by mediating the growth factor-induced activation of receptor tyrosine kinases (15, 16). NG2/CSPG4 can interact with collagen VI, and this NG2/CSPG4-Col VI interplay may regulate interaction between soft-tissue sarcoma cells and the tumor microenvironment (17). Interestingly, driving oncogenic mutations in *Ng2/Cspg4*-expressing cells results in the formation of sarcomas (18).

Altered *NG2/CSPG4* expression and/or distribution may serve as a prognostic factor in various cancer types (19–23). In soft-tissue sarcomas, *NG2/CSPG4* expression is correlated with tumor progression (24, 25). Inhibition of *NG2/CSPG4* expression or treatment with anti-NG2/CSPG4 antibodies inhibits tumor growth in xenografts from some malignancies (26–28). However, the efficacy of targeted NG2/CSPG4 therapy has not been investigated in sarcomas. Here, we use genetically modified mice, human tumors established as xenografts in mice, and an NG2/CSPG4 antibody-based therapy to study the role of *Ng2/Cspg4* in soft-tissue sarcoma initiation and growth *in vivo*.

## Results

### Deletion of Ng2/Cspg4 in soft-tissue sarcoma reduces tumor size and cell proliferation

**Figure 1.**
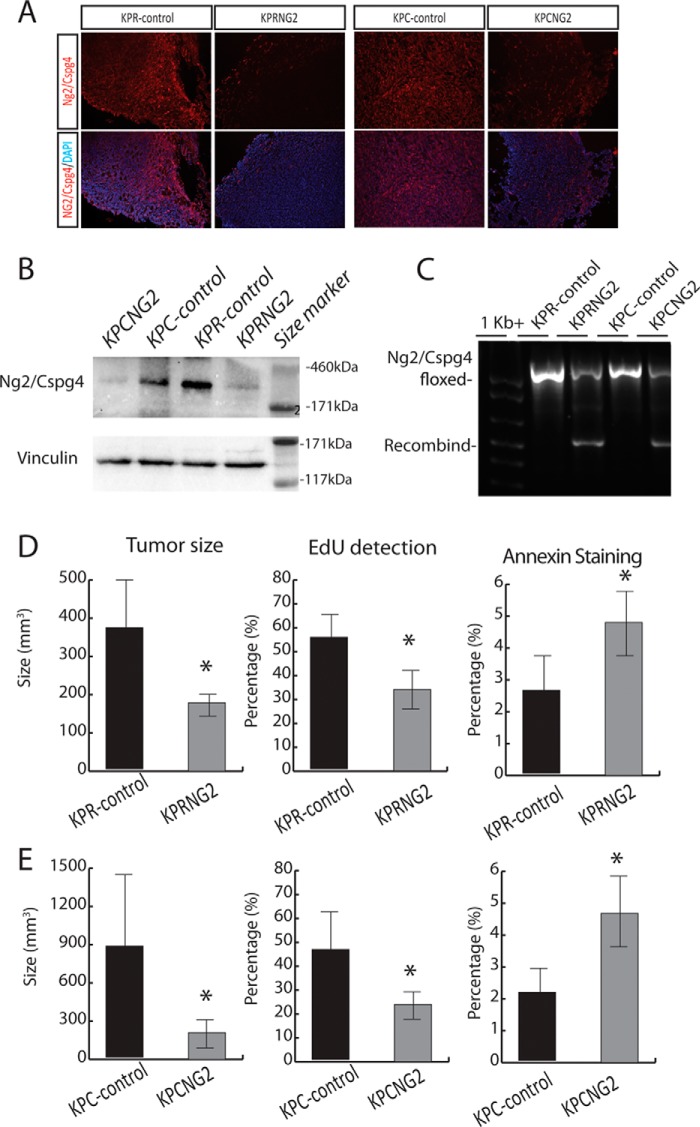
**Ng2/Cspg4 regulates soft-tissue sarcoma tumor growth.** *A*, immunofluorescence staining for Ng2/Cspg4 in KPR-control, KPRNG2, KPC-control, and KPCNG2 tumor samples showing >80% deletion of Ng2/Cspg4 in KPRNG2 tumors and ∼65% deletion of Ng2/Cspg4 in KPCNG2 tumors. *B*, Ng2/Cspg4 protein levels in KPR-control, KPRNG2, KPC-control, and KPCNG2 tumors were assessed by Western analysis, and vinculin was used as loading control. *C*, genotyping of tumors derived from KPR-control, KPRNG2, KPC-control, and KPCNG2 tumors showing recombination at the *Ng2/Cspg4* locus in KPRNG2 and KPCNG2 tumors. A representative blot is shown. *D* and *E*, tumor size and proliferation was significantly decreased, and apoptosis increased in KPRNG2 and KPCNG2 tumors compared with KPR-control and KPC-control tumors, respectively. Data are shown with 95% confidence intervals indicated (*error bars*). *n* = 14 in the KPRNG2 and control groups and 15 in the KPCNG2 and control group. *, *p* < 0.05. The percentage of EdU-positive cells was found in KPRNG2 tumors compared with KPR-control tumors (*n* = 6 in each group), and percentage of annexin V–stained cells (*n* + 6 in each group). Data are shown as means with 95% confidence intervals indicated. *, *p* < 0.05.

We used an autochthonous mouse model in which undifferentiated soft-tissue sarcoma formation is initiated by deletion of both p53 alleles and expression of an oncogenic mutant Kras driven by either Flp recombinase or Cre recombinase (KP mouse). Previous studies show that when the conditional alleles are activated by local injection of a virus driving expression of Flp or Cre in muscle, this results in tumors with characteristics similar to those of human undifferentiated pleomorphic sarcomas (18, 29, 30). To determine the role *Ng2/Cspg4* plays in sarcoma tumor growth and maintenance, we employed a dual recombinase system by crossing *Ng2/Cspg4^f/f^* mice with *Kras*^*FRT-STOP-FRT-G12D*/+^*; p53^FRT/FRT^; Rosa26*^*Cre-ER-T2*/+^ mice to generate *Kras*^*FRT-STOP-FRT-G12D*/+^*; p53^FRT/FRT^; Rosa26*^*Cre-ER-T2*/+^*; Ng2/Cspg4^f/f^* mice (*KPRNG2*). Primary sarcomas were generated in the hind limbs of these mice by intramuscular injection of adeno-FlpO. After the initial tumor was palpated, tamoxifen (200 μg/g) was delivered via intraperitoneal injection. Tumors from *Kras*^*FRT-STOP-FRT-G12D*/+^*; p53^FRT/FRT^; Rosa26*^+/+^*; Ng2/Cspg4^f/f^* (*KPR-control*) and *Kras*^*FRT-STOP-FRT-G12D*/+^*; p53^FRT/FRT^; Rosa26*^*Cre-ER-T2*/+^*; Ng2/Cspg4^f/f^* mice were collected 12 days after tumor formation, and real-time PCR, immunofluorescence, and Western analysis (Fig. 1 (*A* and *B*) and Fig. S1) were used to confirm the deletion of *Ng2/Cspg4* and its protein product. Immunofluorescence showed a 65% reduction in the proportion of cells expressing NG2/CSPG4 in KPCNG2 mice and an 80% reduction in KPRNG2 mice. Western analysis showed a relative NG2/CSPG4 protein level of 14% compared with controls in tumors from KPCNG2 mice and 8% compared with controls in tumors from KPRNG2 mice (relative densities are compared using Student's *t* test, *n* = 5 in each group, *p* < 0.01). We also confirmed the recombination at the *Ng2/Cspg4* locus in the *Kras*^*FRT-STOP-FRT-G12D*/+^*; p53^FRT/FRT^; Rosa26*^*Cre-ER-T2*/+^; *Ng2/Cspg4^f/f^* tumors by PCR analysis of genomic DNA (Fig. 1*C*). Deletion of *Ng2/Cspg4* in established tumors (*Kras*^*FRT-STOP-FRT-G12D*/+^*; p53^FRT/FRT^; Rosa26*^*Cre-ER-T2*/+^; *Ng2/Cspg4^f/f^*) resulted in a significant reduction in tumor size when compared with the *Kras*^*FRT-STOP-FRT-G12D*/+^*; p53^FRT/FRT^; Rosa26*^+/+^; *Ng2/Cspg4^f/f^* tumors (Fig. 1*D*).

Because the *Rosa26*^*Cre-ER-T2*/+^ allele is expressed in tumor cells and non-tumor cells, the results using the *Rosa26*^*Cre-ER-T2*/+^ mice may be due to a non-sarcoma cell effect of NG2/CSPG4 regulating the niche to alter tumor behavior. Therefore, we generated mice in which *Ng2/Cspg4* would be deleted only in the tumor cells. To achieve this, we crossed *Ng2/Cspg4^f/f^* mice with *Kras^FRT-STOP-FRT-G12D^; p53^FRT/FRT^; Col1a1*^*FRT-STOP-FRT-Cre-ER-T2*/+^ mice to generate *Kras^FRT-STOP-FRT-G12D^; p53^FRT/FRT^; Col1a1*^*FRT-STOP-FRT-Cre-ER-T2*/+^*; Ng2/Cspg4^f/f^* (*KPCNG2*) mice. In *Kras^FRT-STOP-FRT-G12D^; p53^FRT/FRT^; Col1a1^FRT-STOP-FRT-Cre-ER-T2^* mice, in which Cre-ER^T2^ is downstream from a *FRT-STOP-FRT* cassette, cells will only express Cre-ER^T2^ and have the capacity for tamoxifen-mediated recombination of *loxP* sites after FlpO-mediated removal of the STOP cassette. Therefore, we utilized *Col1a1*^*FRT-STOP-FRT-Cre-ER-T2*/+^ mice for sequential mutagenesis restricted to the sarcoma cells to investigate the role of tumor-specific *Ng2/Cspg4* in tumor maintenance. Sarcomas were generated in the hind limbs of these mice by intramuscular injection of adeno-FlpO. After the initial tumor was palpated, a single dose of 0.75 mg of 4-hydroxytomaxifen (4-OHT)^2^ in DMSO was delivered via intratumoral injection. Tumors were collected 12 days after the first day of tumor detection. Because complex genetic mice do not always exhibit the expected degree of recombination, we confirmed that *Cre-ER^T2^* was expressed in sarcomas, but not control tissues, using real-time PCR. We then investigated the degree of recombination at the *Ng2/Cspg4* locus in the *Kras^FRT-STOP-FRT-G12D^; p53^FRT/FRT^; Col1a1*^*FRT-STOP-FRT-Cre-ER-T2*/+^*; Ng2/Cspg4^f/f^* tumors by PCR analysis of genomic DNA (Fig. 1*C*) and used immunofluorescence and Western analysis to assess deletion at the protein level (Fig. 1*B*). Tumors from *Kras^FRT-STOP-FRT-G12D^; p53^FRT/FRT^; Col1a1*^*FRT-STOP-FRT-Cre-ER-T2*/+^*; Ng2/Cspg4^f/f^* mice showed partial deletion of *Ng2/Cspg4* expression compared with *Kras*^*FRT-STOP-FRT-G12D*/+^*; p53^FRT/FRT^; Rosa26*^*Cre-ER-T2*/+^; *Ng2/Cspg4^f/f^* tumors. This degree of deletion resulted in a significant reduction in tumor size when compared with tumors from *Kras^FRT-STOP-FRT-G12D^; p53^FRT/FRT^; Col1a1*^+/+^*; Ng2/Cspg4^f/f^* mice (Fig. 1*E*). Thus, deletion of *Ng2/Cspg4* in the neoplastic cells themselves reduced the soft-tissue sarcoma tumor size.

Cell proliferation was analyzed by culturing cells derived from tumors with medium containing 5-ethynyl-2′-deoxyuridine (Edu) for 4 h to label cells in S phase. Tumor cells derived from both sets of mice in which *Ng2/Cspg4* were deleted (*KPRNG2* and *KPCNG2*) and their respective control tumors, in which *Ng2/Cspg4* was expressed, were studied. There was a lower percentage of Edu-positive cells in tumors lacking *Ng2/Cspg4* expression than in controls in both genotypes (Fig. 1, *D* and *E*), showing that Ng2/Cspg4 positively regulates tumor cell proliferation. Apoptosis was determined using annexin V staining, as detected using flow cytometry. There was an increase the proportion of annexin V staining in the absence of *Ng2/Cspg4* expression (Fig. 1, *D* and *E*) in both genotypes as well. Thus, *Ng2/Cspg4* regulates both cell proliferation and apoptosis in soft-tissue sarcomas.

### Knockdown of Ng2/Cspg4 delays xenograft growth

**Figure 2.**
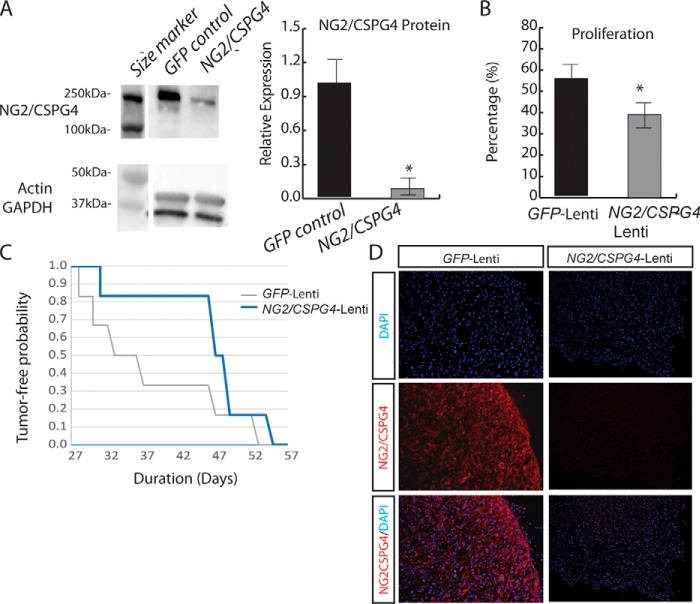
**NG2/CSPG4 regulates human soft-tissue sarcoma cell proliferation and tumor engraftment.** *A*, protein levels of NG2/CSPG4 in GFP-lentivirus or NG2/CSPG4-lentivirus–infected human soft-tissue sarcoma cells were assessed by Western analysis. Actin and GAPDH were used as loading controls. A significant reduction in the level of NG2/CSPG4 expression was found in cells infected with NG2/CSPG4-lentivirus compared with the GFP-lentivirus–infected control cells. *n* = 6 in each group. The Western image is from a single blot with additional lanes not relevant to this figure replaced by a *space* in this image. *B*, knockdown of NG2/CSPG4 resulted in a decrease in the percentage of EdU-positive cells. *n* = 6 in each group. Data are shown as means with 95% confidence intervals indicated (*error bars*). *, *p* < 0.05. *C*, representative Kaplan–Meier curve demonstrated delayed tumor engraftments with cells (1 × 10^4^ cells) infected with lentivirus expressing siRNA to NG2/CSPG4 when compared with the ones with GFP-lentivirus–infected control cells. *n* = 15 in each group. *D*, immunofluorescence staining of human NG2/CSPG4 in xenografted tumors showing 90% deletion of NG2/CSPG4 in NG2/CSPG4-lentivirus–infected tumors compared with GFP-lentivirus–infected tumors.

To determine whether *NG2/CSPG4* in human soft-tissue tumors plays a similar role, we knocked down *NG2/CSPG4* expression in xenografted human tumor cells using a lentivirus expressing shRNA to *NG2/CSPG4*. Infection of human primary undifferentiated pleomorphic sarcoma cells with a lentivirus expressing shRNA to *NG2/CSPG4* resulted in substantial down-regulation of *Ng2/CSPG4* expression, as compared with cells transfected with a control *GFP*-lentivirus (Fig. 2*A*). In cell cultures, there was a decrease in cell proliferation, as measured by the percentage of EdU-positive cells in *NG2/CSPG4*-lentivirus–infected cells, compared with that of the same cells transfected with a control lentivirus (Fig. 2*B*). Lentivirus-infected human undifferentiated pleomorphic sarcoma cells were established as xenografts in NSG mice at serial dilutions ranging from 1 × 10^5^ to 1 × 10^2^ cells/injection. Within this range of number of cells injected, knockdown of *NG2/CSPG4* did not affect the tumor-initiating potential of the sarcoma cells (Table S1). However, we found that cells infected with the lentivirus expressing shRNA to *NG2/CSPG4* lentivirus showed a delay in engraftment at all four dilutions tested (Fig. 2*C*). Histological analyses of explanted tumors in which *NG2/CSPG4* was knocked down showed an indistinguishable morphology from controls (Fig. S2). To verify *NG2/CSPG4* knockdown in the xenografts, an antibody specific for human NG2/CSPG4 was used for immunofluorescent staining analysis in xenograft tumors derived from *NG2/CSPG4*-lentivirus–infected cells and *GFP*-lentivirus–infected cells. Xenografts of human tumor cells that had been infected with *NG2/CSPG4*-lentivirus showed much less NG2/CSPG4 expression (Fig. 2*D*). Because of the difference in time to engraftment, at any given time, the tumors derived from *GFP*-lentivirus–infected cells were smaller in size. However, the cause for this change in tumor size cannot be distinguished between a delay in engraftment and changes in cell proliferation, as the lentiviral construct was not inducible and, as such, could not be activated after engraftment.

### NG2/CSPG4 antibody treatment inhibits the growth of human undifferentiated pleomorphic sarcoma xenografts in SCID mice

**Figure 3.**
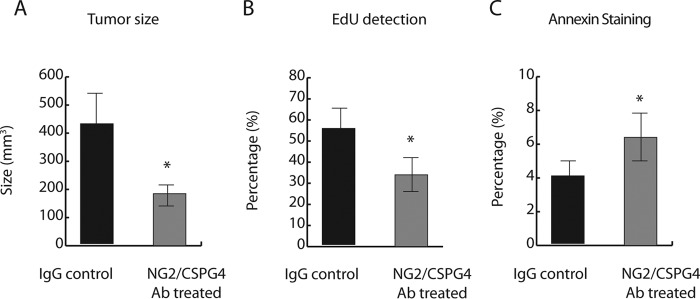
**NG2/CSPG4-antibody immunotherapy inhibits tumor growth in human undifferentiated pleomorphic sarcoma xenografts.** *A*, NG2/CSPG4-antibody treatments resulted in a decrease in tumor weight when compared with tumors treated with IgG; *n* = 10 in each group. *B*, antibody treatment decreased tumor cell proliferation; *n* = 6 in each group. *C*, antibody treatment increased tumor cell apoptosis; *n* = 6 in each group. Data are shown as means with 95% confidence intervals indicated (*error bars*). *, *p* < 0.05.

NG2/CSPG4 has been used as an immunotherapy target in xenografts in melanoma, triple-negative breast cancer, and malignant mesothelioma (27, 59, 60). We thus explored targeting NG2/CSPG4 protein with mAb-based immunotherapy in soft-tissue sarcomas. Human undifferentiated pleomorphic sarcomas xenografted into NSG mice were treated with 50 μg/ml/mouse of either mAb 9.2.27 (Abcam) or isotype control IgG every other day for 2 weeks via intraperitoneal injection. Treatment with mAb 9.2.27 significantly reduced the average tumor volume as compared with IgG controls (Fig. 3*A*). No signs of toxicity were detected in mice treated with mAb 9.2.27. In addition, treatment with mAb 9.2.27 resulted in a decrease in cell proliferation and increase in cell apoptosis (Fig. 3, *B* and *C*). Data here suggest that NG2/CSPG4 mAb–based immunotherapy could be developed into an approach for the treatment of NG2/CSPG4-expressing soft-tissue sarcomas.

### Deletion of Ng2/Cspg4 at the time of tumor initiation resulted in an increase in tumor size

Lentiviral knockdown of *NG2/CSPG4* expression in human tumors established as xenografts delayed engraftment. Because *Ng2/Cspg4* is expressed in mesenchymal precursor cells and could play a role in regulating stem cell-like properties, and driving oncogenic mutations in *Ng2/Cspg4* expressing cells results in sarcoma formation (18), we investigated the role of *Ng2/Cspg4* in tumor initiation in the mouse. *Ng2/Cspg4^f/f^* mice (32) were crossed with *Kras*^*G12D*/+^*; p53^f/f^* mice to generate - *Kras^G12D^; p53^f/f^; Ng2/Cspg4^f/f^* (*KPNG2^f/f^*) mice. Primary tumors were induced with adenovirus expressing Cre-recombinases and harvested 12 days after the initial detection of the tumors. Deletion efficiency was confirmed using real-time PCR, Western blot analysis, and immunofluorescence staining (Fig. S3). Nine of 11 of the *Kras*^*G12D*/+^*; p53^f/f^; Ng2/Cspg4^f/f^* mice developed tumors after the adeno-Cre injection. Whereas this proportion is lower than observed in tumors expressing wildtype *Ng2/Cspg4*, because these sarcomas lacked *Ng2/Cspg4* expression, we know that *Ng2/Cspg4* is not required for tumor initiation.

**Figure 4.**
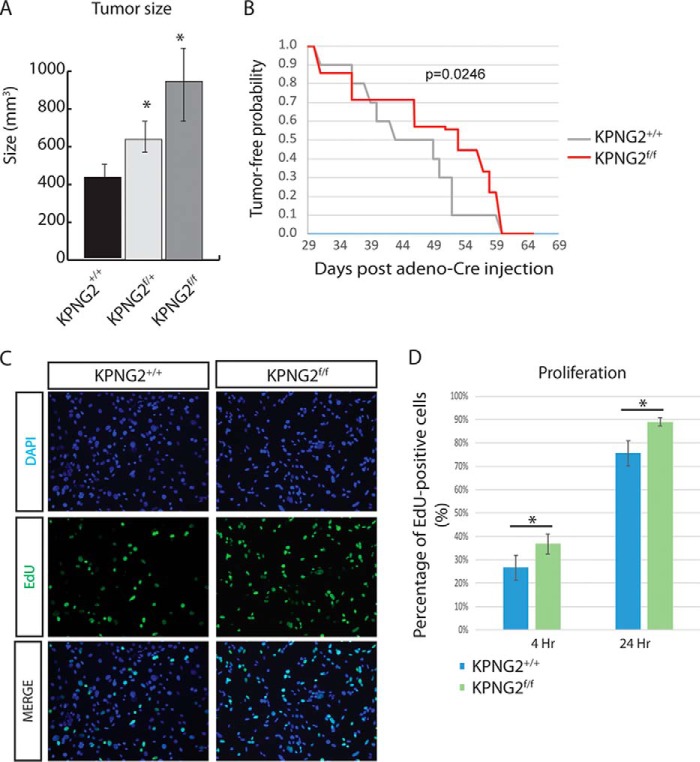
**Deletion of Ng2/Cspg4 at the time of tumor initiation increases tumor size.** *A*, tumor size was significantly larger in KPNG2^f/f^ tumors compared with KPNG2^f/+^ or KPNG2^+/+^ tumors. Data are shown as means with 95% confidence intervals (KPNG2^+/+^, *n* = 8; KPNG2^f/+^, *n* = 17; KPNG2^f/f^, *n* = 9. *B*, KPNG2^f/f^ mice showed a delay in tumor formation compared with KPNG2^+/+^ animals. *p* = 0.0246. *C*, representative images of immunofluorescence staining of EdU showing increased percentage of Edu-positive cells (green channel) in the KPNG2^f/f^ tumor cells compared with KPNG2^+/+^ tumor cells after 4 h of culturing with medium containing 10 μm Edu. Nuclei were labeled with 4′,6-diamidino-2-phenylindole (*DAPI*) staining (*blue channel*). *D*, percentage of EdU-positive cells were analyzed after 4 and 24 h of culturing with EdU-containing medium. Increased percentages of EdU-positive cells were found at both time points in the KPNG2^f/f^ cells compared with KPNG2^+/+^ cells. Data are shown as means with 95% confidence intervals indicated (*error bars*). *, *p* < 0.05.

Interestingly, tumors from the *Kras*^*G12D*/+^*; p53^f/f^; Ng2/Cspg4^f/f^* mice were significantly larger than the ones from either *Kras*^*G12D*/+^*; p53^f/f^; Ng2/Cspg4*^+/+^ or *Kras*^*G12D*/+^*; p53^f/f^; Ng2/Cspg4*^*f*/+^ mice (Fig. 4*A*), and they formed at a similar time following injection as tumors in control mice expressing *Ng2/Cspg4* (Fig. 4*B*). When cells derived from these sarcomas were cultured in Edu-containing medium, there was increased proliferation in the *Kras*^*G12D*/+^*; p53^f/f^; Ng2/Cspg4^f/f^* tumor cells compared with *Kras*^*G12D*/+^*; p53^f/f^; Ng2/Cspg4*^+/+^ cells (Fig. 4, *C* and *D*) as detected by EdU staining.

### Ng2/Cspg4 has divergent effects on gene expression, depending on the developmental stage of the tumor when it is depleted

To investigate an explanation behind the divergent effect of *Ng2/Cspg4* deletion on tumor behavior at different stages, we compared RNA expression profiles between tumors expressing *Ng2/Cspg4* and those in which *Ng2/Cspg4* was deleted either at the time of tumor initiation or after a tumor formed. RNA sequencing was compared between tumors from *Kras*^*G12D*/+^*; p53^f/f^; Ng/Cspg4*^+/+^, *Kras*^*G12D*/+^*; p53^f/f^; Ng/Cspg4^f/f^, Kras*^*FRT-STOP-FRT-G12D*/+^*; p53^FRT/FRT^; Rosa26*^+/+^; *Ng2/Cspg4^f/f^* and *Kras*^*FRT-STOP-FRT-G12D*/+^*; p53^FRT/FRT^; Rosa26*^*Cre-ER-T2*/+^; *Ng2/Cspg4^f/f^* mice. The data have been deposited in the GEO database (accession number GSE97489).

**Figure 5.**
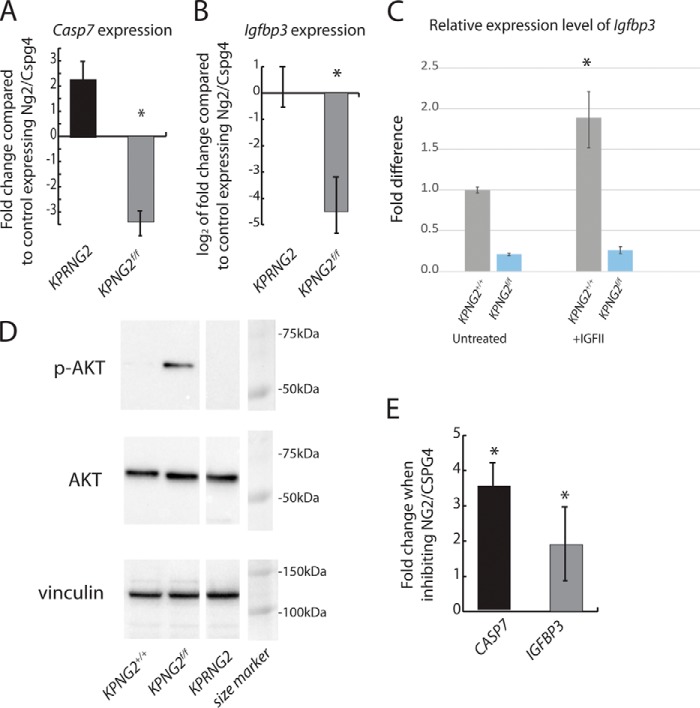
**Divergent effect of Ng2/Cspg4 deletion on gene expression, depending on the developmental stage of the tumor when it is depleted.** *A*, -fold change of Casp7 expression, showing an increase when *Ng2/Cspg4* is deleted after the tumor forms but a decrease when deleted at the time of tumor initiation compared with control tumors expressing *Ng2/Cspg4. n* = 6 for each condition. *B*, -fold change of *Igfbp3* expression, showing a decrease when *Ng2/Cspg4* is deleted after the tumor forms but an increase when deleted at the time of tumor initiation compared with control tumors expressing *Ng2/Cspg4. n* = 6 for each condition. Expression is reported as log_2_. *C*, KPNG2^+/+^ and KPNG2^f/f^ tumor cells were cultured with IGFII (100 ng/ml) for 12 h followed by real-time PCR analysis for *Igfbp3* expression. Data are shown as means with 95% confidence intervals indicated. *n* = 6 for each group. *, *p* < 0.05. *D*, increased expression levels of phospho-AKT were found in the KPNG2^f/f^ tumors when compared with tumors derived from KPNG2^+/+^ or KPRNG2 animals. Vinculin was used as loading control. The Western image is from a single blot with additional lanes not relevant to this figure replaced by a *space* in this image. *E*, change in expression of *CASP7* and *IGFBP3* in a human tumor treated with an NG2/CSPG4 compared with controls (control = 1). There is a significant increase in expression in both genes. Data are shown as means with 95% confidence intervals indicated (*error bars*). *, *p* < 0.05.

When *Ng2/Cspg4* is depleted after a tumor has formed, there is increased expression of genes implicated in the regulation of apoptosis (*e.g. Casp7*), consistent with our findings of increased annexin V staining in these lesions. This was confirmed by real-time PCR (Table S2 and Fig. 5*A*). When *Ng2/Cspg4* is depleted at the time of tumor initiation, many of these genes are down-regulated (Table S3 and Fig. 5*A*). Interestingly, there was a significant down-regulation of several *Igfbp* genes in the *Kras^G12D^; p53^f/f^; Ng2/Cspg4^f/f^* tumors when compared with either *Kras^G12D^; p53^f/f^; Ng2/Cspg4*^+/+^ or *Kras*^*FRT-STOP-FRT-G12D*/+^*; p53^FRT/FRT^; Rosa26*^*Cre-ER-T2*/+^; *Ng2/Cspg4^f/f^* tumors (Tables S3 and S4). The down-regulation of *Igfbp3* was validated using quantitative real-time PCR (Fig. 5*B*).

Because *Igfbp3* was substantially down-regulated in the *Kras*^*G12D*/+^*; p53^f/f^; Ng2/Cspg4^f/f^* tumors but not in *Kras*^*FRT-STOP-FRT-G12D*/+^*; p53^FRT/FRT^; Rosa26*^*Cre-ER-T2*/+^; *Ng2/Cspg4^f/f^* tumors, its regulation of expression could not be due to changes in Ng2/Cspg4 alone. Because IGFII-mediated IGF signaling activation up-regulates *Igfbp3* expression (33), its down-regulation could be due to the loss of sensitivity to IGFII. We thus examined Ng2/Cspg4 regulation of *Igfbp3* regulation upon IGFII stimulation *in vitro*. Tumor cells derived from *Kras*^*G12D*/+^*; p53^f/f^; Ng2/Cspg4*^+/+^, and *Kras*^*G12D*/+^*; p53^f/f^; Ng2/Cspg4^f/f^* mice were treated with IGFII followed by real-time PCR analysis for *Igfbp3* expression. Treatment of IGFII up-regulates *Igfbp3* expression in *Kras*^*G12D*/+^*; p53^f/f^; Ng2/Cspg4*^+/+^ tumor cells, but this stimulatory effect is lost in the *Kras*^*G12D*/+^*; p53^f/f^; Ng2/Cspg4^f/f^* tumor cells (Fig. 5*C*).

Igfbp3 can function as a pro-apoptotic factor to decrease the survival cancer cells (34–37). IGFBPs also down-regulate IGF signaling (38), and IGF signaling can activate AKT to affect tumor cell viability (39, 40). Thus, deletion of *Ng2/Cspg4* at the time of tumor initiation could result in an increase in tumor size by promoting tumor cell proliferation through down-regulation of *Igfbp3.* In support of this notion, a link between Ng2/Cspg4 and AKT activity was shown in lung cancer cells (41). Thus, we examined the AKT activity in the tumor cells derived from *Kras*^*G12D*/+^*; p53^f/f^; Ng2/Cspg4*^+/+^, and *Kras^G12D^; p53^f/f^; Ng2/Cspg4^f/f^* mice using Western analysis. We found a significant increase in the level of phospho-AKT in the *Kras*^*G12D*/+^*; p53^f/f^; Ng2/Cspg4^f/f^* cells when compared with the *Kras^G12D^; p53^f/f^; Ng2/Cspg4*^+/+^ cells (Fig. 5*D*). To determine whether the findings in the mouse correlated with the situation in human tumors, we examined the expression of *CAS7* and *IGFBP3* in tumors treated with NG2/CSPG4 antibody and found increased expression in both genes with inhibition (Fig. 5*E*). Thus, the human tumors behave similarly to the mouse tumors in which *Ng2/Cspg4* is depleted in an established tumor. Taken together, these data raise the possibility that deleting *Ng2/Cspg4* at tumor initiation activates IGF signaling, a pathway known to positively regulate soft-tissue sarcoma growth (42), whereas in established tumors, there is an opposite effect.

## Discussion

Here we show that *Ng2/Cspg4* plays an important role in soft-tissue sarcoma growth and maintenance. Inhibition of Ng2/Cspg4 in established sarcomas by gene deletion or NG2/CSPG4 antibody immunotherapy significantly reduced tumor size in murine and human sarcomas. This was associated with a decrease in cell proliferation and increase in apoptosis.

Surprisingly, deleting *Ng2/Cspg4* at the time of tumor initiation resulted in the opposite effect on tumor growth. We found that deletion of *Ng2/Cspg4* at tumor initiation resulted in activation of IGF signaling and a loss of the normal regulation of insulin growth factor–binding proteins, which are known to inhibit IGF signaling and regulate cell proliferation. It is possible that cells might adapt to loss of *Ng2/Cspg4* at tumor initiation in ways that are different from *Ng2/Cspg4* after the tumor is already formed. This adaptation can include activation of IGF signaling through the loss of normal regulation of IGFBPs. This might be related to an epigenetic change, allowing activation of IGF signaling through the loss of normal up-regulation of IGFPBs. Another possibility is that because Ng2/Cspg4 is a membrane proteoglycan that interacts with other cells and extracellular matrix, loss of *Ng2/Cspg4* at tumor initiation may alter a critical extracellular interaction that promotes tumor growth. Deletion of *Ng2/Cspg4* at tumor initiation revealed a pro-apoptotic effect of Ng2/Cspg4, so ablation of *Ng2/Cspg4* might account for decreased cell death at the time of tumor initiation. Consistent with that notion, Ng2/Cspg4 has been shown to play a pro-apoptotic role in fibroblasts (43, 44). Another possibility is that by deleting *Ng2/Cspg4* at tumor initiation, we are changing the cell type that becomes the tumor or changing the type of tumor that develops; however, in our analysis to date, we have not found differences between the sarcomas to suggest a difference in tumor cell type. Our data on phospho-AKT, and a lack of normal down-regulation of *Igfbp3* expression, suggest that there is constitutive IGF activity in the tumors that lost *Ng2/Cspg4* at tumor initiation. This adds support to the notion that IGF plays a critical role in tumor cell behavior in sarcomas (45, 46).

Soft-tissue sarcoma comprises a heterogeneous group of tumors; therefore, precise categorization of patient tumor types is important for choosing appropriate and effective treatments. Our data suggest that targeting Ng2/Cspg4 can be developed into a novel therapeutic approach for soft-tissue sarcomas expressing this transmembrane proteoglycan. Furthermore, our data also illustrate the complexity in interpreting results from genetically engineered mice, as the data from deletion of *Ng2/Cspg4* at tumor initiation probably do not necessarily pertain to the more clinically relevant experiment of *Ng2/Cspg4* inhibition in established tumors. Issues related to experiments studying the role of genes in tumor initiation compared with their role in tumor maintenance should be considered when translating data from mouse genetics to proposed therapies.

## Experimental procedures

### Mice

All experiments were performed in accordance with National Institutes of Health guidelines and were approved by the Duke University Division of Laboratory Resources. We crossed *Ng2/Cspg4*^−/−^, *Ng2/Cspg4^f/f^* (32, 47), *LSL-Kras^G12D^; p53^f/f^* (29), *Kras^FRT-STOP-FRT-G12D^; p53^FRT/FRT^* (48, 49), and *R26^Cre-ER-T2^* or *Col1a1^FRT-STOP-FRT-Cre-ER-T2^* transgenic mice. Tumors were generated by intramuscular injection of a calcium phosphate precipitate of adenovirus expression Cre recombinase or flippase (University of Iowa) into the right hind leg of 6–8-week-old mice. Animals were euthanized within 12 days of tumor formation. Volume was calculated by using the formula, (*ab*^2^)π/6, where *a* is the longest measurement and *b* is the shortest.

Tomoxifen-induced genes were activated using 4-OHT (Sigma-Aldrich) dissolved in 100% ethanol at a concentration of 250 mg/ml and then diluted with DMSO (Sigma-Aldrich) to a final working solution concentration of 25 mg/ml. Soft-tissue sarcomas in the mouse hind limb were infiltrated with a single intramuscular injection of 30 μl of 4-OHT (0.75 mg) working solution via a 27.5-gauge insulin syringe.

### NG2/CSPG4 lentiviral particle transfection and human sarcoma xenografts

Human undifferentiated pleomorphic sarcoma cells were dissociated into single cells as described previously (50, 51). Lentiviral particles containing three target-specific constructs that encode 19–25-nucleotide shRNA targeting human *NG2/CSPG4* and lentiviral particles expressing copGFP control lentiviral particles were purchased from Santa Cruz Biotechnology. Cells were plated 24 h before viral transfection at 0.6 × 10^5^ cells/well. For cell infection, viral particles (multiplicity of infection = 4) were supplemented with 10 μg/ml Polybrene and incubated with cells for 24 h. Puromycin (2 μg/ml) was used to select stable clones expressing the shRNA. Various numbers of cells (1 × 10^5^ to 1 × 10^2^) were suspended with Matrigel (BD Biosciences) and injected subcutaneously into 6–8-week-old NSG mice (Jackson Laboratory).

### Histology and immunofluorescence

Tumor samples were formalin-fixed in 4% paraformaldehyde, embedded in Tissue Tek O.C.T compound (Fisher Scientific), and sectioned. Sections (10 μm) of the tumor samples were stained with hematoxylin and eosin following standard procedures. For immunofluorescence, the sections were stained with antibodies against human NG2/CSPG4 (mouse mAb 9.2.27, 1:100) or mouse Ng2/Cspg4 (rabbit anti-Ng2/EC, 1:100) at 4 °C overnight. Secondary antibodies conjugated with Alexa Fluor 488 (1:1000; Invitrogen) or Alexa Fluor 568 (1:1000; Thermo Fisher Scientific) were incubated for 1 h at room temperature to detect the primary antibody. Finally, the sections were mounted in 4′,6-diamidino-2-phenylindole containing Fluoroshield (Abcam) and imaged.

### Western analysis

Western analysis was performed using standard protocols. Immunoblotting was performed overnight at 4 °C with the following primary antibodies: human NG2/CSPG4 mAb 9.2.27 (1:1000), mouse Ng2/Cspg4 (1:2000), phospho-AKT (Ser-473) from Cell Signaling (1:1000), pan-AKT from Cell Signaling (1:1000), actin from Thermo Fisher (1:5000), and vinculin from Millipore (1:2000).

### Gene expression analysis

RNA isolated from at least three independent experiments was analyzed by quantitative real-time PCR in triplicate for each treatment condition and primer set. The reactions used TaqMan Universal PCR master mix (Applied Biosystems) with TaqMan gene expression assays for human *NG2/CSPG4*, mouse *Ng2/Cspg4*, and mouse *Igfbp3* (Applied Biosystems). The gene expression levels between samples were analyzed using the 2^ΔΔ^*^Ct^* method (52). *Gapdh* (Applied Biosystems) was used as endogenous control for target gene normalization. 50 ng of total RNA was used for cDNA synthesis using RT^2^ SYBR Green quantitative PCR master mixes and the RT^2^ First Strand kit (Qiagen). Gene expression profiling was performed using the RT^2^ Profiler Mouse Cancer PathwayFinder PCR Array (Qiagen) according to the manufacturer's protocol.

### RNA sequencing

RNA-seq data were processed using the TrimGalore toolkit, which employs Cutadapt to trim low-quality bases and Illumina sequencing adapters from the 3′-end of the reads. Only reads that were 20 nucleotides or longer after trimming were kept for further analysis. Reads were mapped to the GRCm38v68 version of the mouse genome and transcriptome (53) using the STAR RNA-seq alignment tool (54). Reads were kept for subsequent analysis if they mapped to a single genomic location. Gene counts were compiled using the HTSeq tool. Only genes that had at least 10 reads in any given library were used in subsequent analysis. Normalization and differential expression was carried out using the DESeq2 (55) Bioconductor (56) package with the R statistical programming environment. The false discovery rate was calculated to control for multiple hypothesis testing. Gene set enrichment analysis (57) was performed to identify differentially regulated pathways and gene ontology terms for each of the comparisons performed.

### Proliferation and apoptosis

Cell proliferation was measured using the Click-iT EdU imaging kit (Invitrogen) according to the manufacturer's protocols. Cells isolated from sarcomas were cultured with medium containing 10 μm Edu for 4 or 24 h followed by fixation and EdU detection. Apoptosis was measured using annexin V incorporation in a manner identical to that previously reported (58).

### Immunotherapy studies

Human undifferentiated pleomorphic sarcoma cells (1 × 10^4^) were suspended with Matrigel (BD Biosciences) and injected subcutaneously into 6–8-week-old NSG mice. After 7 weeks, the animals were randomly divided into two groups. Animals were treated with mouse monoclonal antibody 9.2.27 to human NG2/CSPG4 or IgG control at 50 μg/ml/mouse (Abcam) via intraperitoneal injection every other day for 2 weeks.

### Statistical analyses

For all of the data, the mean, 95% confidence interval, and S.D were calculated for each condition. Statistical significance was calculated using two-tailed, unpaired Student's *t* test. The threshold for statistical significance was *p* < 0.05.

## Author contributions

S.-C. H., W. B. S., D. G. K., and B. A. A. designed experiments, interpreted results, and wrote the manuscript. S.-C. H. performed genetic and cell culture experiments. P. N. and V. P. performed the xenograft immunotherapy experiment.

## Supplementary Material

Supporting Information

## Footnotes

2 The abbreviations used are:

4-OHT: 4-hydroxytomaxifen
Edu: 5-ethynyl-2′-deoxyuridine
IGF: insulin-like growth factor.
