# Supplementary material for: Effects of chondroitin sulfate proteoglycan 4 (NG2/CSPG4) on soft-tissue sarcoma growth depend on tumor developmental stage
Source: J Biol Chem. 2017 Dec 1;293(7):2466–75. doi: 10.1074/jbc.M117.805051 (PMC5818183; doi:10.1074/jbc.M117.805051)
Supplement: Supporting Information [file supp_293_7_2466__index.html]

Effects of chondroitin sulfate proteoglycan 4 (NG2/CSPG4) on soft tissue sarcoma growth depend on tumor developmental stage — Effects of chondroitin sulfate proteoglycan 4 (NG2/CSPG4) on soft-tissue sarcoma growth depend on tumor developmental stage — NG2/CSPG4 in sarcomas — Supporting Information 

# Effects of chondroitin sulfate proteoglycan 4 (NG2/CSPG4) on soft-tissue sarcoma growth depend on tumor developmental stage

## Supporting Information

- supplemental data (.pdf, 7.5 MB) - supplemental data
