# Supplementary material for: Effects of chondroitin sulfate proteoglycan 4 (NG2/CSPG4) on soft-tissue sarcoma growth depend on tumor developmental stage
Source: J Biol Chem. 2017 Dec 1;293(7):2466–75. doi: 10.1074/jbc.M117.805051 (PMC5818183; doi:10.1074/jbc.M117.805051)
Supplement: Supporting Information [file 10.1074_M117.805051_jbc.M117.805051-1.pdf]

Supplementary data

Supplemental Tables

Supplemental Table 1

Number of cells required to form a xenograft tumor from a human undifferentiated pleomorphic sarcoma infected with a construct expressing a lentivirus expressing an anti- NG2/CSPG4 construct or a control. Knockdown of NG2/CSPG4 does not increase the number of cells required to form a xenograft tumor.

	Cell number injected	Tumors/Sites injected	Percentage
GFP-lentivirus	1×10^5	4/4	100%
	1×10^4	6/6	100%
	1×10^3	6/6	100%
	1×10^2	4/4	100%
anti-NG2/CSPG4-lentivirus	1×10^5	4/4	100%
	1×10^4	6/6	100%
	1×10^3	6/6	100%
	1×10^2	4/4	100%

Supplemental Table 2

Differential expression between tumors in which *Ng2/Cspg4* is depleted after a tumor has formed with those expressing *Ng2/Cspg4*. Statistically significant greater than twofold changes are shown from tumors that developed in $Kras^{FRT\text{-}STOP\text{-}FRT\text{-}G12D/+}$; $p53^{FRT/FRT}$; $Rosa26^{+/+}$; $Ng2/Cspg4^{f/f}$ mice compared to tumors from $Kras^{FRT\text{-}STOP\text{-}FRT\text{-}G12D/+}$; $p53^{FRT/FRT}$; $Rosa26^{Cre\text{-}ER\text{-}T2/+}$; $Ng2/Cspg4^{f/f}$ mice. The full data set is deposited in the GEO database (GEO: GSE97489).

Gene Symbol	Fold regulation
Adm	-7.9357
Casp7	2.2036
Ccl2	-4.8336
Cdc20	2.6256
Cox5a	2.3761
Dkc1	4.199
E2f4	2.9902
Ets2	2.6077
Fgf2	-2.6991
Foxc2	2.3001
Gadd45g	3.3183
Gpd2	2.1287
Hmox1	-3.7693
Igfbp3	-2.4944
Igfbp5	10.1147
Igfbp7	-3.7891
Ldha	-2.0909
Lig4	2.3335
Lpl	-2.6735
Mcm2	2.4923
Pfkl	-2.9899
Pgf	44.0699
Serpinb2	-4.1965
Serpinf1	-3.9415
Sirt1	2.6702
Skp2	3.2384
Stmn1	2.1397
Terf2ip	2.2312
Tnks2	2.0209
Uqcrfs1	2.3196
Vegfc	2.2911
Xrcc4	2.3267
Hsp90ab1	2.5509

Supplemental Table 3

Differential expression between tumors in which *Ng2/Cspg4* is depleted at the time of tumor initiation with those expressing *Ng2/Cspg4*. Statistically significant greater than twofold changes are shown from tumors that developed in *$Kras^{G12D}$; $p53^{f/f}$; $Ng2/Cspg4^{f/f}$* mice compared to tumors from *$Kras^{G12D}$; $p53^{f/f}$; $Ng2/Cspg4^{+/+}$* mice. The full data set is deposited in the GEO database (GEO: GSE97489).

**Gene Symbol**	**Fold regulation**
Acs14	-3.34
Adm	-16.20
Angpt1	5.10
Car9	-2.24
Casp7	-3.12
Casp9	-2.98
Ccl2	-8.94
Ccnd3	-4.26
Cdh2	-2.49
Cflar	-2.80
Ets2	-2.28
Fgf2	-7.50
Gadd45g	-9.00
Gpd2	-2.09
Gsc	-8.12
Hmox1	-5.85
Igfbp3	-358.35
Igfbp5	-233.07
Igfbp7	-16.31
Kdr	-3.26
Lpl	3.95
Map2k1	-3.58
Map2k3	-3.33
Nol3	-19.98
Pfk1	-2.82

Pgf	-20.34
Serpinb2	-37.20
Serpinf1	-2.38
Slc2a1	-3.44
Snail	-2.68
Tek	-3.66
Tep1	-3.71
Vegfc	-11.95

Supplemental Table 4

Differential expression between tumors in which *Ng2/Cspg4* is depleted at tumor initiation with tumors in which *Ng2/Cspg4* is depleted after a tumor has formed. Statistically significant greater than twofold changes are shown from tumors that developed in from $\textit{Kras}^{G12D}$*; *$\textit{p53}^{f/f}$*; *$\textit{Ng2/Cspg4}^{f/f}$ mice compared to tumors from $\textit{Kras}^{FRT\text{-}STOP\text{-}FRT\text{-}G12D/+}$*; *$\textit{p53}^{FRT/FRT}$*; *$\textit{Rosa26}^{Cre\text{-}ER\text{-}T2/+}$; $\textit{Ng2/Cspg4}^{f/f}$ mice. The full data set is deposited in the GEO database (GEO: GSE97489).

**Gene Symbol**	**Fold regulation**
Acs14	-3.86
Angpt1	4.69
Atp5a1	-2.07
Car9	-4.15
Casp7	-7.19
Casp9	-2.40
Ccl2	-3.14
Ccnd3	-3.79
Cdc20	-2.15
Cdh2	-3.56
Cflar	-2.02
Cox5a	-2.60
Cpt2	-2.01
Dsp	2.47
E2f4	-2.37

Ercc3	-2.18
Ets2	-2.70
Fgf2	-5.50
Flt1	5.41
Gadd45g	-43.91
Gpd2	-2.44
Gsc	-3.04
Hmox1	-3.06
Igfbp3	-74.02
Igfbp5	-251.18
Igfbp7	-10.01
Lig4	-2.91
Lpl	3.06
Map2k1	-2.81
Map2k3	-3.27
Mcm2	-2.35
Nol3	-48.83
Pgf	-337.10
Serpinb2	-17.75
Sirt1	-3.68
Sirt2	-2.03
Slc2a1	-3.01
Stmn1	-2.91
Terf2ip	-3.64
Vegfc	-13.12
Weel	-2.09
Xrcc4	-2.56
Actb	-2.82
Hsp90ab1	-2.32

Supplementary figures and legends

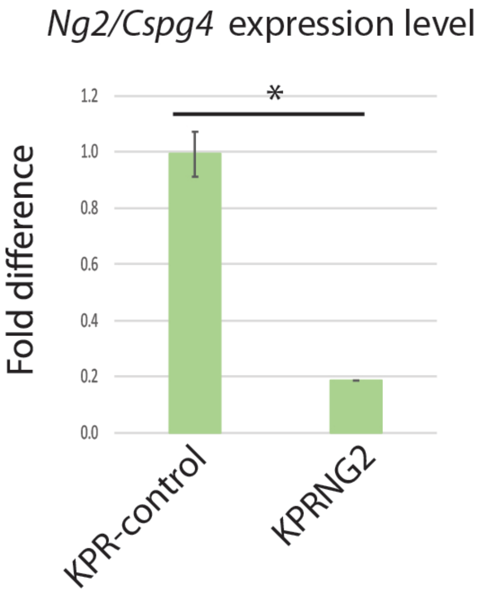

Supplemental Figure One

Supplemental Figure 1: Level of *Ng2/Cspg4* expression in tumors generated from *Kras*$^{FRT\text{-}STOP\text{-}FRT\text{-}G12D/+}$; *p53*$^{FRT/FRT}$; *Rosa26*$^{Cre\text{-}ER\text{-}T2/+}$; *Ng2/Cspg4*$^{+/+}$ (KPR-control) mice or *Kras*$^{FRT\text{-}STOP\text{-}FRT\text{-}G12D/+}$; *p53*$^{FRT/FRT}$; *Rosa26*$^{Cre\text{-}ER\text{-}T2/+}$; *Ng2/Cspg4*$^{f/f}$ (KPRNG2) mice.

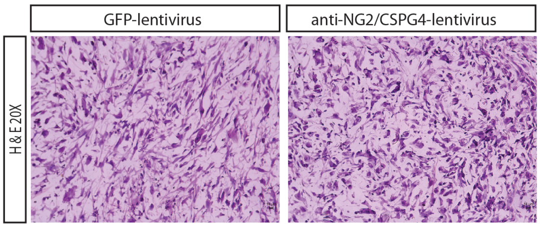

Supplemental Figure Two

Supplemental Figure 2: Histology of tumors that formed from human undifferentiated pleomorphic sarcoma cells established as xenografts infected with and anti-NG2/CSPG4 construct or control. No significant difference is observed in cytology between control and NG2/CSPG4 knockdown.

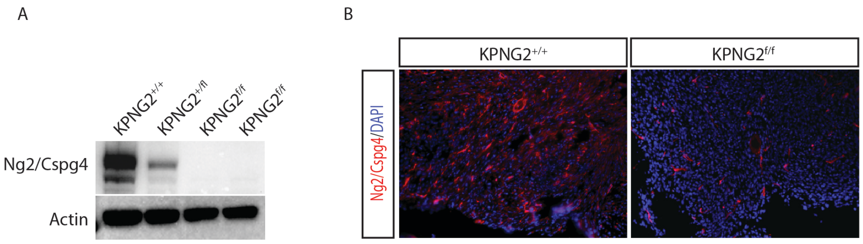

Supplemental Figure Three

Supplemental Figure 3: Deletion efficiency for NG2/CSPG4 protein was confirmed by Western blot analysis (Panel A) , and Immunofluorescence staining (panel B) for tumors from *Kras*^{G12D}*; p53*$^{f/f}$*; Ng2/Cspg4*$^{f/f}$ (*KPNG2*$^{f/f}$) mice.
